# Effect of ultrashort-acting β-blockers on 28-day mortality in patients with sepsis with persistent tachycardia despite initial resuscitation: a meta-analysis of randomized controlled trials and trial sequential analysis

**DOI:** 10.3389/fphar.2024.1380175

**Published:** 2024-06-20

**Authors:** Po Huang, Fusheng Liu, Xiao Hu, Bo Li, Xiaolong Xu, Qingquan Liu

**Affiliations:** ^1^ Beijing Dongfang Hospital, Beijing University of Traditional Chinese Medicine, Beijing, China; ^2^ Beijing Hospital of Traditional Chinese Medicine, Affiliated with Capital Medical University, Beijing, China; ^3^ Beijing Institute of Traditional Chinese Medicine, Beijing, China

**Keywords:** ultrashort-acting β-blockers, mortality, sepsis, tachycardia, meta-analysis

## Abstract

**Purpose:**

This meta-analysis aims to identify whether patients with sepsis who have persistent tachycardia despite initial resuscitation can benefit from ultrashort-acting β-blockers.

**Materials and methods:**

Relevant studies from MEDLINE, the Cochrane Library, and Embase were searched by two independent investigators. RevMan version 5.3 (Cochrane Collaboration) was used for statistical analysis.

**Results:**

A total of 10 studies were identified and incorporated into the meta-analysis. The results showed that the administration of ultrashort-acting β-blockers (esmolol/landiolol) in patients with sepsis with persistent tachycardia despite initial resuscitation was significantly associated with a lower 28-day mortality rate (risk ratio [RR], 0.73; 95% confidence interval [CI], 0.57–0.93; and *p*˂0.01). Subgroup analysis showed that the administration of esmolol in patients with sepsis was significantly associated with a lower 28-day mortality rate (RR, 0.68; 95% CI, 0.55–0.84; and *p*˂0.001), while there was no significant difference between the landiolol and control groups (RR, 0.98; 95% CI, 0.41–2.34; and *p* = 0.96). No significant differences between the two groups were found in 90-day mortality, mean arterial pressure (MAP), lactate (Lac) level, cardiac index (CI), and troponin I (TnI) at 24 h after enrollment.

**Conclusion:**

The meta-analysis indicated that the use of esmolol in patients with persistent tachycardia, despite initial resuscitation, was linked to a notable reduction in 28-day mortality rates. Therefore, this study advocates for the consideration of esmolol in the treatment of sepsis in cases where tachycardia persists despite initial resuscitation.

## Introduction

Currently, sepsis remains a life-threatening condition in emergency and critical care medicine due to its dysregulated inflammatory response to infection (Singer M et al., 2016; Hollenberg and Steven, 2023). Despite early active bundle treatment, mortality rates remain high (Li et al., 2022; Weng et al., 2023). Traditionally, β-blockers have been viewed as having negative effects on myocardial inotropy and the heart rate, potentially impacting hemodynamics and reducing cardiac output in sepsis patients. This has led to debates regarding the use of β-blockers in this patient population. However, recent research studies (Parker et al., 1987; Kuipers et al., 2014; Walkey et al., 2014; Hayase et al., 2016) have indicated that tachycardia significantly increases sepsis mortality, and a reduction in the heart rate (within 24 h) can lead to improved outcomes (Parker et al., 1987). As a result, there is a growing interest in studying the effectiveness of β-blockers in managing septic tachycardia (Walkey et al., 2014; Yang et al., 2023).

Previous research has demonstrated the effectiveness of esmolol in achieving target heart rates and reducing the 28-day mortality of septic patients with tachycardia (Morelli et al., 2013). Subsequent meta-analyses have further supported the benefits of esmolol in sepsis/septic shock patients (Liu et al., 2018; Huang et al., 2021). The potential therapeutic advantages of landiolol, a highly selective β1 receptor blocker, in septic patients with tachycardia warrant additional investigation (Lall et al., 2021; Gangl et al., 2022). A recent multicenter clinical trial (J-Land 3S) revealed that although landiolol did not decrease the 28-day mortality of septic patients, it did significantly lower their heart rates, allowing more patients to reach the desired 60–94 beats/min range within 24 h without an increase in adverse events (Kakihana et al., 2020). However, another multicenter clinical trial (Whitehouse et al., 2023) found that administering landiolol to sepsis patients did not decrease the SOFA score but increased the 28-day mortality and adverse event rates. Consequently, the use of landiolol in sepsis patients is not recommended. Therefore, it is crucial to systematically assess the efficacy of esmolol and landiolol in sepsis. Hasegawa et al. (2021) addressed this, but they evaluated esmolol and landiolol together without a separate analysis of landiolol.

A significant concern about the use of β1-blockers for sepsis is the potential decrease in cardiac output, which may result in hypotension. Therefore, the treatment of tachycardia in septic shock remains a topic of controversy. There is ongoing debate regarding the suitability of both esmolol and landiolol for patients with sepsis and tachycardia, given that they are both β1-blockers. This study aims to evaluate esmolol and landiolol as distinct interventions to determine their impact on the prognosis of sepsis patients with tachycardia.

## Materials and methods

This study adhered to the recommendations and checklist outlined in the Preferred Reporting Items for Systematic Review and Meta-Analysis (PRISMA) statement (Moher et al., 2009). The protocol was registered in PROSPERO (www.crd.york.ac.uk) on 15 January 2024 (Registration ID: CRD42024497520).

### Search strategy

The databases PubMed, Embase, and the Cochrane Library were searched from their inception to 3 January 2024 using keywords such as esmolol, landiolol, sepsis, and randomized controlled trial (RCT). The theme of word search for tachycardia was initially considered in our search, resulting in 44 items. However, we were concerned that this search strategy might be too narrow and could potentially overlook important clinical trials. To broaden the search scope, we implemented the current search method, ultimately yielding 122 items, as shown in Supplementary Figure S1.

### Eligibility criteria of original studies and study selection

In this study, the inclusion criteria consisted of patients aged 18 years or older with sepsis exhibiting persistent tachycardia despite initial resuscitation (after 24 h of hemodynamic optimization aimed at establishing an adequate circulating blood volume, a mixed venous oxygen saturation higher than 65%, and a mean arterial pressure [MAP] of 65 mmHg or higher, while their heart rate persisted at 95/min or higher), meeting sepsis-1, sepsis-2, or sepsis-3 definitions. The interventions included IV esmolol/landiolol, with the control being a placebo or no intervention. Primary outcomes focused on 28-day mortality, while secondary outcomes included 90-day mortality, heart rate (HR), MAP at 24 h post-enrollment treatment, norepinephrine (NE) dose, lactate level, cardiac index (CI), stroke volume index (SVI), troponin I (TnI), length of stay (LOS) in the ICU, and adverse events. The study design was an RCT, with abstracts and titles screened by two independent reviewers (HX and HP), and any disagreements were resolved through discussion to reach a consensus. The authors of the included trials were contacted for clarification when needed.

### Data extraction

A pre-defined data extraction form was used in this study, with two reviewers (HX and HP) independently extracting information such as the first author, published year, sample size, intervention, control, and outcomes from the selected trials. Any discrepancies between the two reviewers were resolved through discussion with a third reviewer until a consensus was reached.

### Bias risk in individual trials

The methodological quality of the studies was evaluated by independent reviewers (HX and HP) to assess the risk of bias. Any disagreements were resolved through further discussions. In cases where reaching a conclusion was challenging, a third reviewer (XXL) reviewed the entire article and participated in the discussion. The risk of bias in each trial was assessed using the Cochrane Collaboration tool for evaluating bias in randomized trials.

### Statistical analysis

Statistical analysis was conducted using RevMan version 5.3 from the Cochrane Collaboration. The Cochrane Handbook of Systematic Reviews guided the selection of risk ratios (RRs) and 95% confidence intervals (CIs) for dichotomous outcome evaluation, while the mean difference and its 95% CI were used for continuous outcomes. Between-study heterogeneity was assessed using an I^2^ test, where 25% or lower indicated low heterogeneity, 50% indicated moderate heterogeneity, and 75% indicated high heterogeneity. The fixed-effects model was used in cases of no or low heterogeneity, and pooled RRs were calculated using the Mantel–Haenszel method. Publication bias was investigated if there were more than 10 studies for a specific outcome. A significance level of *p* < 0.05 was applied. Trial sequential analysis (TSA) was conducted for subgroup analysis of esmolol using TSA viewer version 0.9 Beta (http://www.ctu.dk/tsa/). The parameters used were as follows: the control group had a mortality rate of 50%, the experimental group had a mortality rate of 35%, a two-sided α = 0.05 for the difference test, and 1-β = 0.8.

## Results

### Description and risk of bias of included studies

**FIGURE 1:**
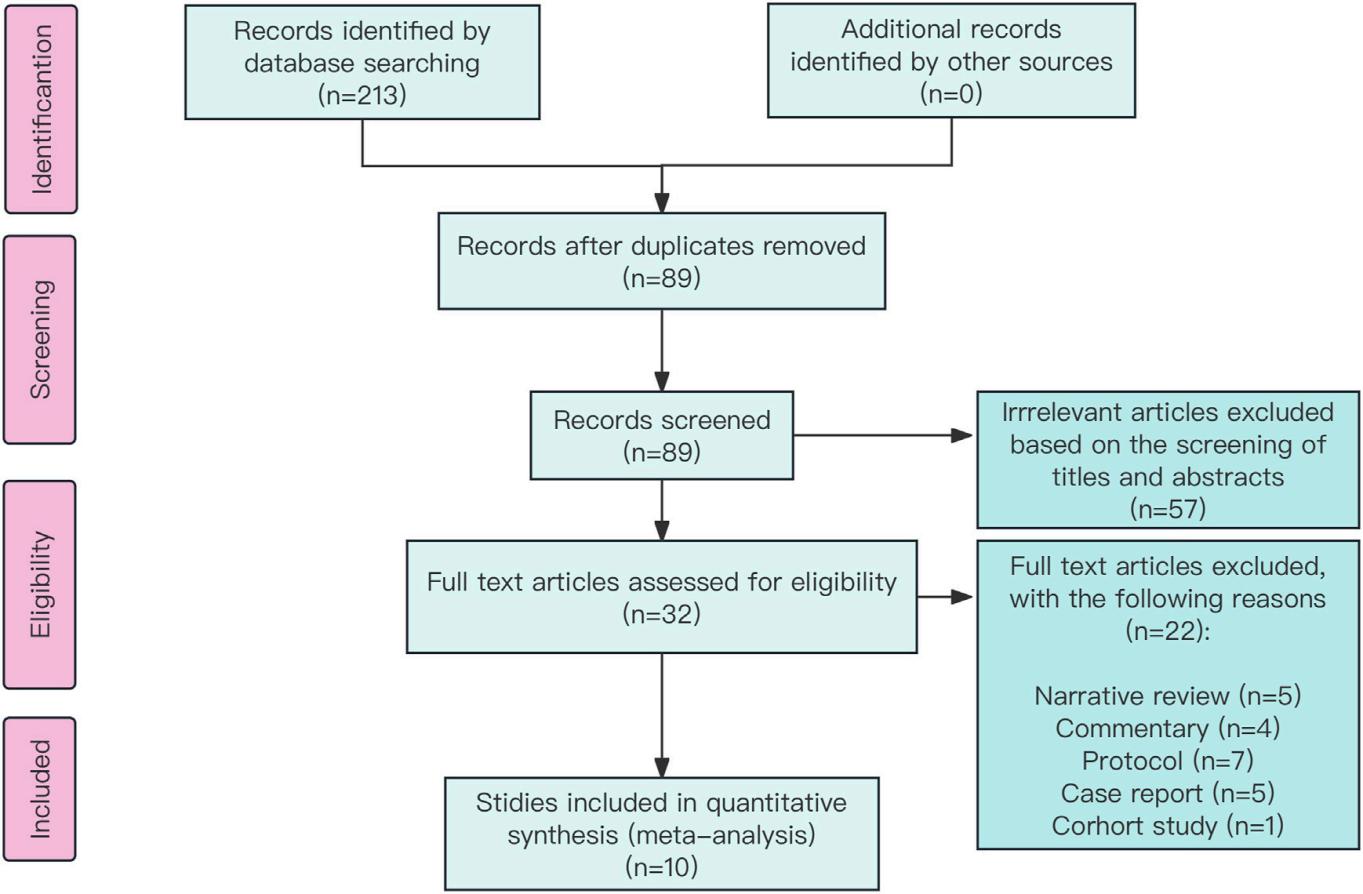
Preferred Reporting Items for Systematic Reviews and Meta-Analyses chart: identification and selection of studies for inclusion.

A total of 213 records were identified using the search strategy, with 89 potentially eligible records obtained after removing duplicates. Following the screening of titles and abstracts, 57 studies were excluded, resulting in the inclusion of 10 studies (Morelli et al., 2013; Yang et al., 2014; Liu et al., 2015; Wang et al., 2015; Wang et al., 2017; Liu et al., 2019; Kakihana et al., 2020; Cocchi et al., 2022; Wang et al., 2023; Whitehouse et al., 2023) involving 881 participants (Figure 1). The characteristics of these included studies are given in Table 1. The risk of bias in these studies was assessed using the Cochrane Collaboration tool, with the results given in Supplementary Figure S2. All 10 studies (Morelli et al., 2013; Yang et al., 2014; Liu et al., 2015; Wang et al., 2015; Wang et al., 2017; Liu et al., 2019; Kakihana et al., 2020; Cocchi et al., 2022; Wang et al., 2023; Whitehouse et al., 2023) were deemed to have low risk in terms of random sequence generation, incomplete outcome data, and selective reporting. Five studies (Liu et al., 2019; Kakihana et al., 2020; Cocchi et al., 2022; Wang et al., 2023; Whitehouse et al., 2023) were considered to have a low risk for allocation concealment, while this could not be assessed in the other five studies (Morelli et al., 2013; Yang et al., 2014; Liu et al., 2015; Wang et al., 2015; Wang et al., 2017) due to insufficient information. One study (Wang et al., 2017) was rated as low risk, while five (Morelli et al., 2013; Wang et al., 2015; Liu et al., 2019; Kakihana et al., 2020; Cocchi et al., 2022) were rated as high risk for blinding of participants and personnel. Similarly, two studies (Kakihana et al., 2020; Cocchi et al., 2022) were deemed high risk and four (Morelli et al., 2013; Wang et al., 2015; Liu et al., 2019; Whitehouse et al., 2023) were deemed low risk for blinding of outcome assessment, with the assessment being inconclusive for the remaining four studies (Yang et al., 2014; Liu et al., 2015; Wang et al., 2017; Wang et al., 2023) due to inadequate information. In terms of other biases, four studies (Yang et al., 2014; Kakihana et al., 2020; Cocchi et al., 2022; Whitehouse et al., 2023) were conducted at multiple centers and considered low risk, while the remaining six studies (Morelli et al., 2013; Liu et al., 2015; Wang et al., 2015; Wang et al., 2017; Liu et al., 2019; Wang et al., 2023) conducted at a single center were deemed high risk.

**TABLE 1:** Characteristics of the included studies.

Study	Country	No. of participants	Intervention		Setting	Study period	Outcomes	Administration of esmolol/landiolol
			Experimental group	Control group				
Morelli et al. (2013)	Italy	154	Esmolol	None	Single center	November 2010 to July 2012	HR, 28-day mortality, hemodynamic markers, norepinephrine dose, and adverse events	Administration of esmolol: initiated after randomization that was performed after resuscitation with fluid and vasopressors for 24 h. Initial dose of esmolol: initiated at 25 mg/h
								Titration or tapering: by ≥ 50 mg/h, slowly every 20 min, to reach HR between 80 and 94 beats/min within 12 h. Duration of administration: esmolol was continued until ICU discharge or death. The permitted maximum dose was 2,000 mg/h
Yang et al. (2014)	China	41	Esmolol	None	Single center	January 2012 to January 2014	Hemodynamic parameters including MAP, CVP, HR, CO, CI, SVI, and SVRI and tissue oxygenation parameters including ScvO_2_ and Lac	Administration of esmolol: initiated after randomization that was performed after 6 h resuscitation with fluid and vasopressors. Initial dose of esmolol: 0.05 mg/kg/min. Titration of tapering: adjusted to achieve HR of <100 beats/min in 2 h. Duration of administration: not reported
Liu et al. (2015)	China	48	Esmolol	None	Single center	September 2013 to September 2014	28-day mortality, LOS in ICU, hemodynamic parameters such as HR, MAP, CVP, CI, SVI, and SVRI and tissue oxygen metabolism markers	Administration of esmolol: initiated after randomization that was performed after resuscitation with fluid and vasopressors for 6 h. Initial dose of esmolol: initiated at 0.05 mg/kg/min. Titration or tapering: adjusted to reach HR of <100 beats/min within 24 h. The permitted maximum dose was not specified. Duration of administration: not reported
Wang et al. (2015)	China	60	Esmolol + milrinone	None	Single center	June 2013 to June 2014	28-day survival rate, HR, hemodynamic and organ function variables, myocardial injury markers, serum pro-inflammatory markers, norepinephrine dose, and adverse events	Administration of esmolol: not reported. Initial dose of esmolol: not reported
								Titration or tapering: not reported. Duration of administration: not reported
Wang et al. (2017)	China	60	Esmolol	None	Single center	August 2014 to October 2016	28-day mortality, cardiac output, MAP, CI, SVI, inflammatory markers, hemodynamics, and organ function markers	Administration of esmolol: initiated after randomization that was performed after resuscitation with fluid and vasopressors for 24 h. Initial dose of esmolol: initiated at 3 mg/kg/min. Titration or tapering: by 50 mg/h every 5 min to reach HR of <95 beats/min within 4 h. The permitted maximum dose was 12 mg/kg/min. Duration of administration: esmolol was continued for 24 h
Liu et al. (2019)	China	100	Esmolol	None	Single center	June 2016 to August 2017	28-day mortality; 7-, 60-, and 90-day mortalities; HR; Lac; inflammatory marker; and length of hospital stay	Administration of esmolol: initiated after randomization that was performed after being resuscitated with fluid and vasopressors for 24 h. Initial dose of esmolol: initiated at 25 mg/h. Titration or tapering: by 50 mg/h every 20 min to reach HR between 80 and 100 beats/min within 12 h. The permitted maximum dose was 0.3 mg/kg/min. Duration of administration: esmolol was continued until day 7, ICU discharge, or death
Kakihana et al. (2020)	Japan	151	Landiolol	None	Multicenter	January 2018 to April 2019	28-day mortality, HR, norepinephrine dosages, and adverse events	Administration of landiolol: landiolol was initiated within 2 h after randomization that was conducted after being resuscitated with fluid and vasopressors. Mean time ± SD from entering ICU to randomization was 15.8 ± 13.4 h in the landiolol group and 13.5 12.6 h in the control group. Initial dose of landiolol: initiated at 1 mg/kg/min. Titration or tapering: by 1 mg/kg/min generally every 15–20 min, until the HR decreased to <95 beats/min. Duration of administration: landiolol was continued for at least 96 h after randomization. It was optional between 96 and 168 h after randomization
Cocchi et al. (2022)	America	42	Esmolol	None	Two centers	January 2015 to December 2019	Inflammatory biomarkers and oxygen consumption (VO_2_) and norepinephrine equivalent dose	Esmolol was titrated to a heart rate of 80–94 per min, starting at 50 mcg/kg/min and subsequently increasing every 20 min in increments of 50 mcg/kg/min (or slower at the discretion of the clinical team) until the target was achieved. The maximum allowed dose was 300 mcg/kg/min. Esmolol was continued for 24 h
Whitehouse et al. (2023)	United Kingdom	126	Landiolol	None	Multicenter	19 April 2018 to 15 December 2021	SOFA score, 28-day mortality, 90-day mortality, length of hospital stay, LOS in ICU, HR, Lac, MAP, and adverse events	Continuous intravenous infusion of landiolol was started at 1.0 mcg/kg/min, increasing every 15 min by a step change of 1.0 mcg/kg/min to reach the target HR of 80–94 bpm with the expectation that this should be within 6 h. While the patient was receiving vasopressor agents (norepinephrine and/or vasopressin), the landiolol infusion was adjusted by step changes of 1.0 mcg/kg/min to maintain the target HR. It was recommended that the landiolol infusion be stopped for at least 12 h before the patient was discharged from the ICU
Wang et al. (2023)	China	100	Esmolol	None	Single center	March 2020 to September 2021	LVEF, HR, 28-day and 90-day mortality, and adverse events	Administration of esmolol: with a loading dose of 0.5 mg/kg, followed by continuous intravenous pumping at 0.05 mg/kg/min for maintenance. If the effect is not favorable after 4 min, administer the loading dose again and increase the maintenance dose by 0.05 mg/kg/min. The maximum maintenance dose can be increased to 0.2 mg/kg/min. Duration of administration: not reported

### Primary outcome

**FIGURE 2:**
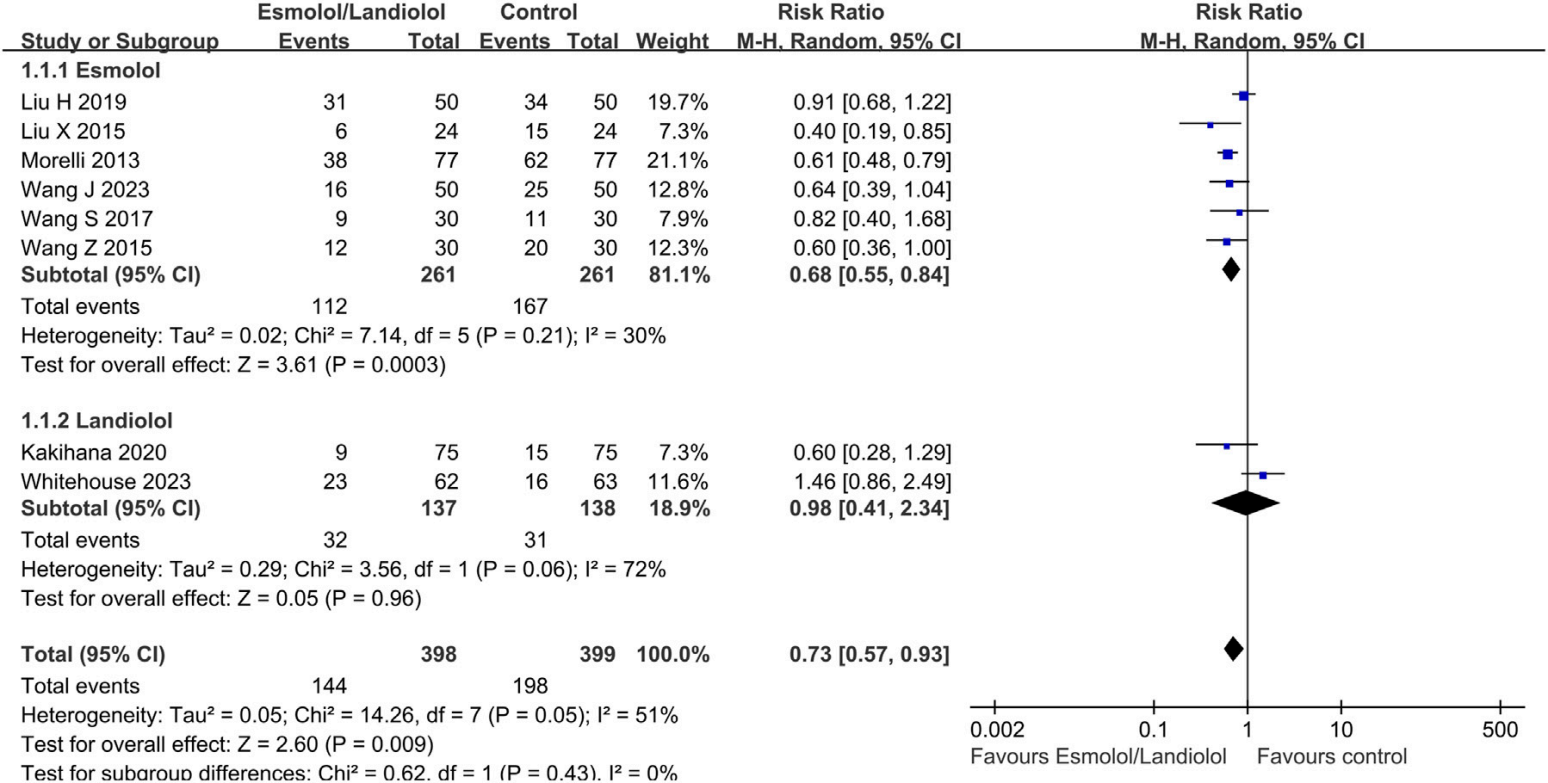
Forest plot of esmolol or landiolol group vs. control group: 28-day mortality. M-H: Mantel–Haenszel.

A total of 8 (Morelli et al., 2013; Liu et al., 2015; Wang et al., 2015; Wang et al., 2017; Liu et al., 2019; Kakihana et al., 2020; Wang et al., 2023; Whitehouse et al., 2023) out of 10 RCTs with 797 participants reported 28-day mortality outcomes. The results indicated that administering ultrashort-acting β-blockers (esmolol/landiolol) to patients with sepsis who had persistent tachycardia despite initial resuscitation was significantly associated with a lower 28-day mortality rate (RR: 0.73; 95% CI: 0.57–0.93; and *p* < 0.01). Notably, subgroup analysis revealed differing outcomes. The use of esmolol in sepsis patients was significantly linked to reduced 28-day mortality (RR: 0.68; 95% CI: 0.55–0.84; and *p* < 0.001), whereas there was no significant difference between the landiolol and control groups (RR: 0.98; 95% CI: 0.41–2.34; and *p* = 0.96) (see Figure 2). The funnel plot is shown in Supplementary Figure S3.

### Secondary outcomes

**FIGURE 3:**
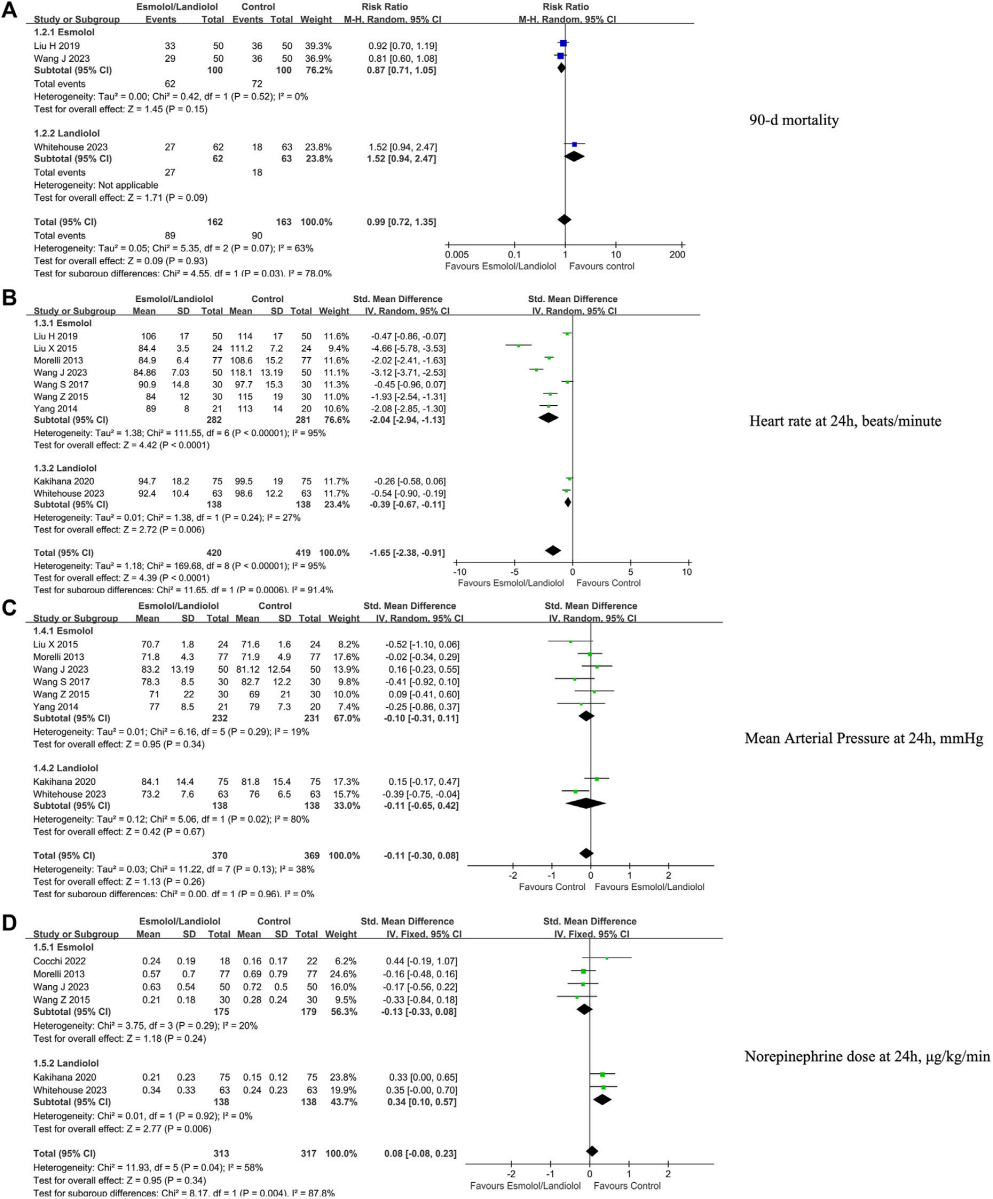
Forest plot of esmolol or landiolol group vs. control group: 90-day mortality, heart rate, MAP, and NE dose at 24 h after enrollment. **(A)** 90-day mortality; **(B)** heart rate at 24 h after enrollment; **(C)** MAP at 24 h after enrollment; and **(D)** NE dose at 24 h after enrollment. MAP, mean arterial pressure; NE, norepinephrine. IV, inverse variance; Std., standardized.

**FIGURE 4:**
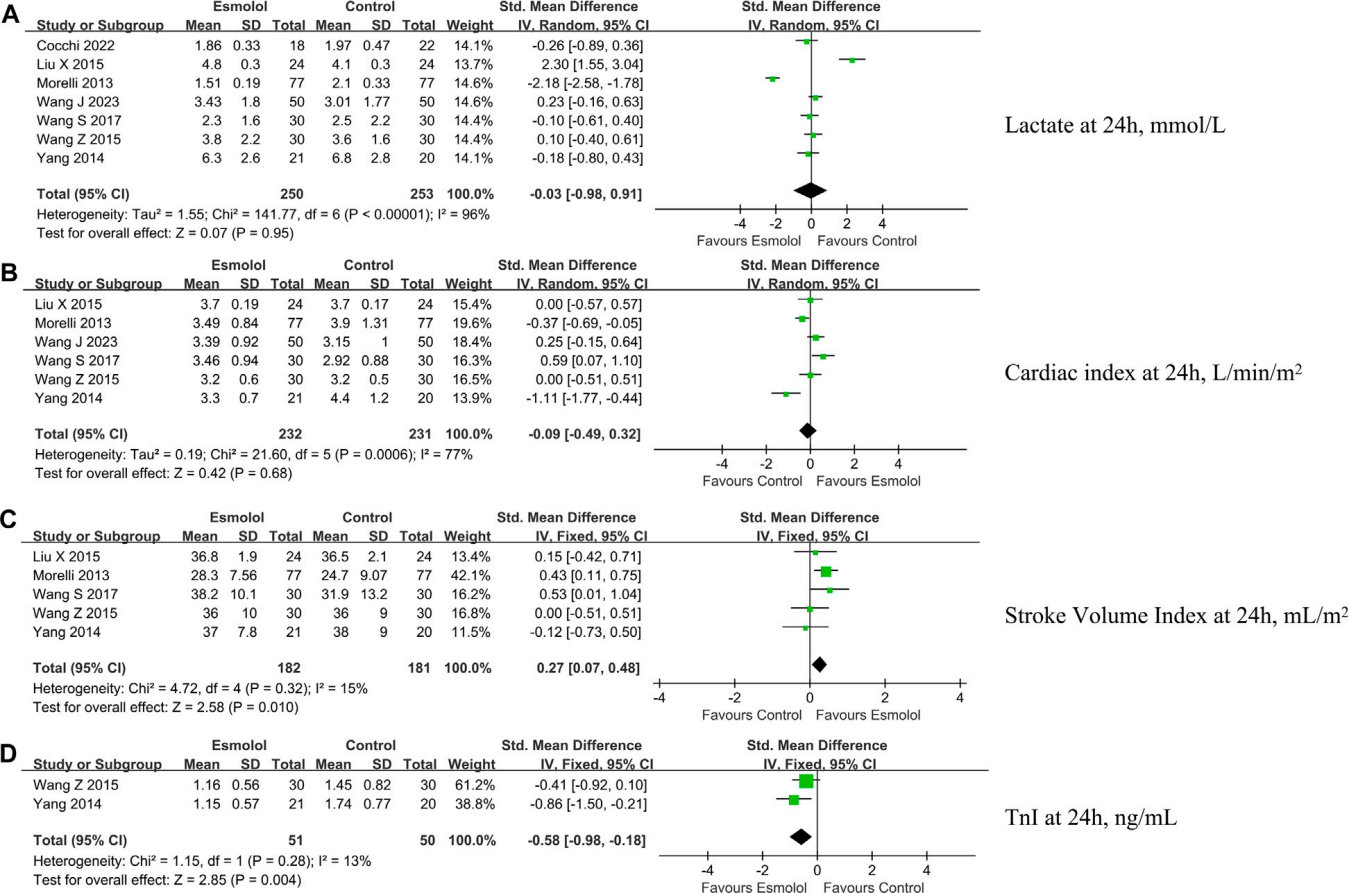
Forest plot of esmolol group vs. control group: Lac, CI, SVI, and TnI at 24 h after enrollment. **(A)** Lac at 24 h after enrollment; **(B)** CI at 24 h after enrollment; **(C)** SVI at 24 h after enrollment; and **(D)** TnI at 24 h after enrollment. Lac, lactate; CI, cardiac index; SVI, stroke volume index. IV, inverse variance; Std., standardized.

**FIGURE 5:**
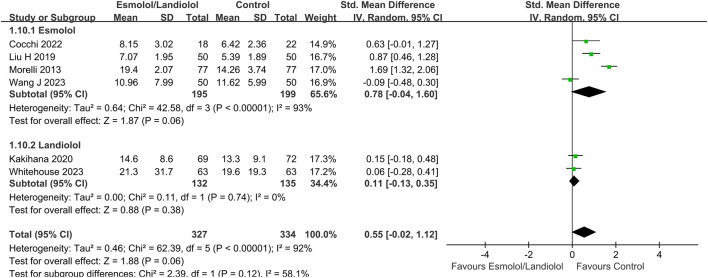
Forest plot of esmolol or landiolol group vs. control group: LOS in ICU. LOS, length of stay; ICU, intensive care unit; IV, inverse variance; Std., standardized.

The study evaluated secondary outcomes including 90-day mortality (Figure 3A), heart rate (Figure 3B), mean arterial pressure (Figure 3C), norepinephrine dose (Figure 3D), lactate levels (Figure 4A), cardiac index (Figure 4B), stroke volume index (Figure 4C), and troponin I levels (Figure 4D) at 24 h after enrollment, and length of stay in the ICU (Figure 5).

Four studies (Morelli et al., 2013; Kakihana et al., 2020; Wang et al., 2023; Whitehouse et al., 2023) examined adverse events, with one (Morelli et al., 2013) showing no adverse events in either group and another reporting asymptomatic bradycardia in the experimental group but no significant arrhythmias in the control group. The remaining two studies (Kakihana et al., 2020; Whitehouse et al., 2023) reported adverse events in both the landiolol and control groups. One study (Kakihana et al., 2020) reported that adverse events were observed in 9 (12%) of the 77 patients in the landiolol group and 8 (11%) of the 74 patients in the control group. Another study (Whitehouse et al., 2023) reported that adverse events were observed in 17.5% (10/63) of those receiving landiolol and 12.7% (8/63) of those receiving standard care.

### Trial sequential analysis

**FIGURE 6:**
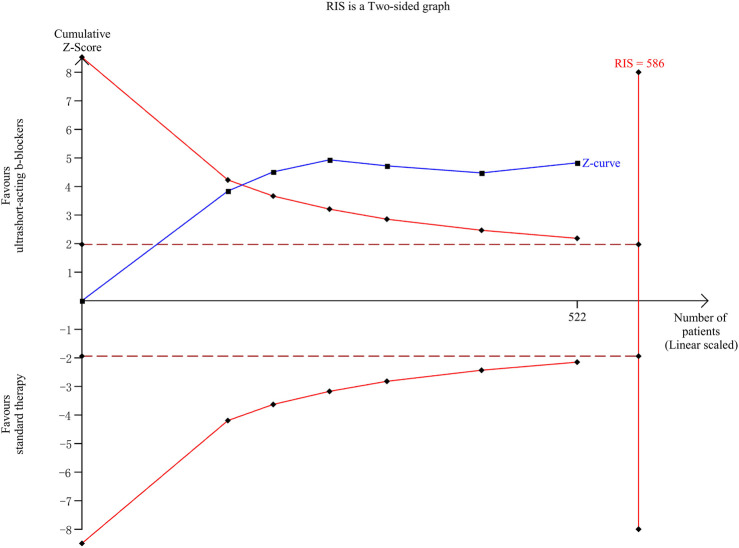
Trial sequential analysis. RIS, required information size. The solid blue line represents the cumulative Z-curve; two symmetrical red solid line curves represent the TSA boundary value; the deep red horizontal dashed line represents the traditional threshold value Z = 1.96; and the red vertical line on the right side represents RIS.

The TSA results are shown in Figure 6. A total of 6 clinical trials (Morelli et al., 2013; Liu et al., 2015; Wang et al., 2015; Wang et al., 2017; Liu et al., 2019; Wang et al., 2023) involving 522 patients were included in the esmolol subgroup meta-analysis, with an actual sample size of 586 cases. The TSA results indicated that the cumulative Z-curve crossed both the traditional and TSA boundary values simultaneously, leading to a positive conclusion being reached before the expected sample size. This suggests that for patients with sepsis who continue to experience tachycardia after initial fluid resuscitation, esmolol provides a 28-day survival advantage with accurate evidence.

## Discussion

The meta-analysis revealed that the use of ultrashort-acting β-blockers (esmolol/landiolol) in septic patients with persistent tachycardia post-resuscitation was linked to reduced 28-day mortality. Subgroup analysis indicated esmolol as the preferred choice for these patients, while the limited sample size prevented the identification of survival benefits with landiolol. This study addresses existing controversies and offers valuable insights for clinical decision-making. The summary of meta-analysis was shown in Table 2.

**TABLE 2:** Summary of meta-analysis.

Outcome	Subgroup	No. of studies	No. of participants	Effect size (95% CI)	*p*
28-day mortality	Total	8	797	RR, 0.73 (0.57, 0.93)	0.009
	Esmolol	6	522	RR, 0.68 (0.55, 0.84)	0.0003
	Landiolol	2	275	RR, 0.98 (0.41, 2.34)	0.96
90-day mortality	Total	3	325	RR, 0.99 (0.72, 1.35)	0.93
	Esmolol	2	200	RR, 0.87 (0.71, 1.05)	0.15
	Landiolol	1	125	RR, 1.52 (0.94, 2.47)	0.09
HR at 24 h	Total	9	839	SMD, −1.65 (−2.38, −0.91)	˂0.0001
	Esmolol	7	563	SMD, −2.04 (−2.94, −1.13)	˂0.0001
	Landiolol	2	276	SMD, −0.39 (−0.67, −0.11)	0.006
MAP at 24 h	Total	8	739	SMD, −0.11 (−0.30, 0.08)	0.26
	Esmolol	6	463	SMD, −0.10 (−0.31, 0.11)	0.34
	Landiolol	2	276	SMD, −0.11 (−0.65, 0.42)	0.67
NE dose at 24 h	Total	6	630	SMD, 0.88 (−0.08, 0.23)	0.34
	Esmolol	4	354	SMD, −0.13 (−0.33, 0.08)	0.24
	Landiolol	2	276	SMD, 0.34 (0.10, 0.57)	0.006
Lac at 24 h	N/A	7	503	SMD, −0.03 (−0.98, 0.91)	0.95
CI at 24 h	N/A	6	463	SMD, −0.09 (−0.49, 0.32)	0.68
SVI at 24 h	N/A	5	363	SMD, 0.27 (0.07, 0.48)	0.01
TnI at 24 h	N/A	2	101	SMD, −0.58 (−0.98, −0.18)	0.004
LOS in ICU	Total	6	661	SMD, 0.55 (−0.02, 1.12)	0.06
	Esmolol	4	394	SMD, 0.78 (−0.04, 1.60)	0.06
	Landiolol	2	267	SMD, 0.11 (−0.13, 0.35)	0.38

Note: HR, heart rate; MAP, mean arterial pressure; NE, norepinephrine; Lac, lactate; CI, cardiac index; SVI, stroke volume index; RR, relative risks; SMD, standard mean difference.

According to the Surviving Sepsis Guideline (Evans et al., 2021), norepinephrine is recommended as a first-line drug for treating septic shock to maintain stable hemodynamics. However, it is well-recognized that norepinephrine use can elevate catecholamine levels, potentially leading to cardiac dysfunction due to sympathetic hyperstimulation (Monnet et al., 2011; Jozwiak, 2022). Studies (Havaldar, 2018; Prescott Hallie and Angus Derek, 2018) have highlighted that cardiac dysfunction plays a crucial role in the poor prognosis of septic patients, with tachycardia increasing cardiac workload and myocardial oxygen consumption, contributing to cardiac dysfunction (Lanspa et al., 2017; Suzuki et al., 2017). Recent research studies have indicated that reducing the heart rate of septic patients to a specific level, alongside adequate fluid resuscitation and circulatory stability, can effectively enhance patient outcomes. A randomized open-label clinical trial conducted by Morelli et al. (2013) and published in JAMA in 2013 yielded positive results. The study demonstrated that all patients in the esmolol group achieved the target heart rate, with a higher cardiac stroke volume index than that in the control group, aligning with our study's findings.

The traditional belief that β-blockers are unsuitable for patients with sepsis/septic shock due to their cardiac suppressive effects is being challenged. This study compared the effects of esmolol and landiolol on the mean arterial pressure, showing no significant difference compared to the control group. However, the landiolol group may require a higher dose of norepinephrine to maintain a relatively stable mean arterial pressure. Both esmolol and landiolol, as selective β1-blockers with a rapid onset of action, require dose titration for optimal bradycardic effects. Landiolol is also approved for treating ventricular fibrillation or tachycardia (Sakamoto et al., 2012; Nagai et al., 2013; Taenaka and Kikawa, 2013). Evidence from case reports, retrospective studies, and animal research supports the use of landiolol for sepsis-related tachyarrhythmias (Okajima et al., 2015; Mathieu et al., 2018; Gangl et al., 2022). Two clinical trials (Kakihana et al., 2020; Whitehouse et al., 2023) specifically evaluated the efficacy of landiolol in sepsis treatment. The J-land 3S study (Kakihana et al., 2020) showed a higher proportion of patients achieving target heart rates in the landiolol group than in the control group, with a significant reduction in new arrhythmias. The results showed that a total of 41 patients (55%) in the landiolol group had a heart rate of 60–94 beats/min 24 h after enrollment, while only 25 patients (33%) in the control group showed the same. There was a statistical difference between the two groups (*p* = 0.0031), and the incidence of new arrhythmias within 7 days was significantly reduced (9% vs. 25%, *p* = 0.015). However, the 28-day mortality rates did not significantly differ between the landiolol and control groups. Another clinical study (Whitehouse et al., 2023) published in JAMA in 2023 (STRESS-L) did not support the use of landiolol in sepsis. The result showed that there was no statistically significant difference between the average SOFA score of the landiolol group (8.8 ± 3.9) and that of the control group (8.1 ± 3.2) (*p* = 0.24). In addition, the 28-day mortality (37.1%) and 90-day mortality (43.5%) rates in the landiolol group were higher than those in the control group (25.4% and 14.9%), but there was no statistical difference between the groups (*p* ˃ 0.05). More importantly, the incidence of serious adverse events in the landiolol group (25.4%) was significantly higher than that of the control group (6.4%), with a statistical difference between the groups (*p* = 0.006).

A previous meta-analysis (Hasegawa et al., 2021) examined the impact of β-blockers on the mortality of patients with sepsis and tachycardia. Our study differs from this previous analysis by conducting a subgroup analysis specifically focusing on the effects of esmolol and landiolol on the mortality of patients with sepsis and tachycardia. Our findings indicate that esmolol significantly reduces the 28-day mortality rate in these patients, whereas landiolol does not show the same effect. Furthermore, the study reveals that landiolol necessitated an increased dose of norepinephrine to maintain mean arterial pressure, suggesting an indirect impact on the hemodynamics of patients with sepsis and tachycardia. Therefore, our study advocates for the use of esmolol in treating patients with septic tachycardia, aligning with findings from previous research. However, the limited sample size prevented the identification of survival benefits with landiolol.

## Conclusion

The meta-analysis indicated that the use of esmolol in patients with persistent tachycardia, despite initial resuscitation, was linked to a notable reduction in 28-day mortality rates. Therefore, this study advocates for the consideration of esmolol in the treatment of sepsis in cases where tachycardia persists despite initial resuscitation.

## Data Availability

The original contributions presented in the study are included in the article/Supplementary Material; further inquiries can be directed to the corresponding authors.
